# Metformin inhibits melanoma cell metastasis by suppressing the miR-5100/SPINK5/STAT3 axis

**DOI:** 10.1186/s11658-022-00353-5

**Published:** 2022-06-15

**Authors:** Dong Suwei, Xiao Yanbin, Wang Jianqiang, Ma Xiang, Peng Zhuohui, Kang Jianping, Wang Yunqing, Li Zhen

**Affiliations:** 1 Department of Orthopaedics, The Second Affiliated Hospital of Xuzhou Medical University, Xuzhou, 221000 People’s Republic of China; 2 Department of Orthopaedics, The Third Affiliated Hospital of Kunming Medical University, Kunming, 650118 People’s Republic of China; 3 Department of Medical Oncology, The Second Affiliated Hospital of Xuzhou Medical University, Xuzhou, 221000 People’s Republic of China; 4 The Second Affiliated Hospital of Xuzhou Medical University, Xuzhou, 221000 People’s Republic of China

**Keywords:** EMT, miR-5100, Metformin, SPINK5, STAT3

## Abstract

Melanoma is the most lethal skin cancer characterized by its high metastatic potential. It is urgent to find novel therapy strategies to overcome this feature. Metformin has been confirmed to suppress invasion and migration of various types of cancer. However, additional mechanisms underlying the antimetastatic effect of metformin on melanoma require further investigation. Here, we performed microarray analysis and uncovered an altered mRNA and miRNA expression profile between melanoma and nevus. Luciferase reporter assay confirmed that miR-5100 targets SPINK5 to activate STAT3 phosphorylation. Migration and wound healing assays showed that the miR-5100/SPINK5/STAT3 axis promotes melanoma cell metastasis; the mechanism was proven by initiation of epithelial–mesenchymal transition. Co-immunoprecipitation (Co-IP) further confirmed an indirect interaction between SPINK5 and STAT3. Furthermore, metformin dramatically inhibited miR-5100/SPINK5/STAT3 pathway, and decreased B16-F10 cell metastasis to lung in C57 mouse module. Intriguingly, pretreatment of metformin before melanoma cell injection improved this effect further. These findings exposed the underlying mechanisms of action of metformin and update the use of this drug to prevent metastasis in melanoma.

**Supplementary Information:**

The online version contains supplementary material available at 10.1186/s11658-022-00353-5.

## Introduction

Cutaneous melanoma is the most aggressive skin cancer derived from melanocytes, accounting for 90% of skin-cancer-related deaths [1, 2] and about one-third of patients diagnosed with metastatic melanoma [3]. Although targeted therapy and immunotherapy has greatly improved objective response rate of patients with advanced melanoma [4], an important subset of patients with melanoma do not respond to these treatments or develop resistance over time [5]. Therefore, it is critical to identify the molecular mechanisms underlying melanoma metastasis.

It has been confirmed that epithelial–mesenchymal transition (EMT) enables the progression and metastasis of melanoma [6]. EMT is a reversible cellular biological process during which epithelial cells lose their polarity features and cell–cell adhesion concomitantly, enabling the cells to acquire motile and invasive features to become mesenchymal cells [7].

MicroRNAs (miRNAs/miRs) are small noncoding RNAs that regulate target genes by recognizing a complementary mRNA sequence and subsequently repressing its translation [8]. A growing number of studies have uncovered the key regulatory role of miRNAs in cancer metastasis [9–11]. On the basis of miRNA assay, we found that miR-5100 expression is increased in melanoma. Moreover, expression profile microarray revealed that serine peptidase inhibitor Kazal type 5 (SPINK5) is decreased in melanoma. SPINK5, a member of the serine protease inhibitor family, has been considered as a tumor suppressor [12, 13]. It is expressed in the stratified epithelial tissues of the skin, and inhibition of SPINK5 leads to enhanced activity of serine protease [14]. Signal transducer and activator of transcription 3 (STAT3) is a critical transcription factor [15]. Studies have suggested that p-STAT3 (Try 705), as the activated form, modulates cancer EMT signals [16], which could be activated by serine proteases [17, 18]. Thus, we suspect that miR-5100 targets SPINK5 and, consequently, activates STAT3.

Metformin (1,1-dimethylbiguanide hydrochloride), a commonly used drug to treat type 2 diabetes, has been found to be associated with a decreased incidence and mortality in several cancers [19]. Moreover, it showed antimetastatic effects in a variety of cancer cells [20]. However, the effect of metformin administration on the EMT process in melanoma has yet to be illuminated.

In the present study, we demonstrated the effects of metformin on the EMT process in melanoma cells. The results showed that metformin inhibited the melanoma EMT process by modulating the miR-5100/SPINK5/STAT3 signaling pathway, suggesting the potential of metformin to serve as an effective antimetastatic drug for melanoma.

## Materials and methods

### Human tissues

Ten formalin-fixed and paraffin-embedded (FFPE) melanoma and nevus samples (five of each) were used for microarrays. For immunohistochemistry staining, we collected another 47 melanoma and 56 nevus tissues from the Third Affiliated Hospital of Kunming Medical University between 2012 and 2018. Of the 47 melanoma and 56 nevus tissues, 18 samples of each group acquired qualified RNA for RT-PCR. All patients received no chemotherapy, radiotherapy, or biotherapy before operation to avoid changes in protein expression resulting from treatment. This study protocol was approved by the Medical Ethics Committee of the Third Affiliated Hospital of Kunming Medical University. Written informed consent was obtained from all patients. The procedures for the collection and use of tissues were performed in accordance with the guidelines of the Declaration of Helsinki, 2013.

### miRNA and mRNA microarray expression analysis

The RecoverAll Total RNA Isolation Kit (Ambion, Canada) was used to extract total RNA (including miRNA) from ten paraffin-embedded melanoma and nevus specimens. Differentially expressed miRNAs were detected using the Agilent Human miRNA Microarray (Agilent V16.0). mRNA expression was detected by Affymetrix microarrays (Affymetrix Almac Xcel Array). Fold change ≥ 2 or ≤ 0.5 was considered as significantly differential expression. The microarray data presented in the present study are available online at the Gene Expression Omnibus of the National Center for Biotechnology Information (NCBI). (https://www.ncbi.nlm.nih.gov/geo/; accession no. GSE183115, GSE183116.

### Cell culture and transfection

293T, human melanoma cell line A2058, G361 and murine melanoma cell line B16-F10 were obtained from the ATCC. The cells were cultured in DMEM medium (Gibco, USA) containing 10% FBS (Gibco, USA). Cells were incubated at 37 °C with 5% CO_2_. siRNA and expression vectors (RiboBio, China) using Lipofectamine® 2000 reagent (Invitrogen, USA) at a final concentration of 50 nM, according to the manufacturer’s instructions. A total of 48 h after transfection, cells were collected for the further experiments. The IC_50_ values were determined as previously reported [21].

### Extraction of RNA and quantitative RT-PCR

Eighteen samples of melanoma and nevus group were employed for RT-PCR (not including samples for microarray test). Total RNA was extracted from cells by using TRIzol (Invitrogen, USA) method according to the kit’s technical manual. Total RNA of the cell was converted into cDNA with miScript Reverse Transcription Kit (Qiagen GmbH), then expression analyses were performed by Roche Lightcycler 480 Real-Time PCR system (Roche Diagnostics, Switzerland) according to the predetermined conditions. Primer sequences and samples clinical parameters are shown in Additional file 1: Data S1. The results were analyzed using the 2^–△△Ct^ method [22]. All samples were analyzed at least in triplicate.

### Western blot analysis

The protein samples were extracted from the cells and tissue was extracted using RIPA buffer (Pierce, USA) and quantitatively measured with a BCA Protein Assay Kit (Thermo, USA). Western blotting was conducted as previously described [23]. Antibodies against SPINK5 (ab138511), STAT3 (ab68153), p-STAT3-Y705 (ab267373), E-cadherin (ab1416), N-cadherin (ab76011), Snail (ab216347), Vimentin (ab92547), and GAPDH (ab181602) were purchased from Abcam (Cambridgeshire, UK).

### Luciferase reporter assay

Melanoma cells were cotransfected with pGL3-basic luciferase reporter vector (wild type or mutant), miR-5100 mimic, or the control. After 48 h of transfection, luciferase assay was performed using the Dual Luciferase Reporter Assay System (Promega, USA) according to the manufacturer’s instructions.

### Wound-healing assays

The cells were seeded into six-well plates, and the wound was scratched by plastic tips when a monolayer formed. To remove the detached cells and debris, the main cells were washed by PBS. The size of the wounds was measured at different timepoints. The experiments were performed at least three times.

### Transwell assays

Transwell migration assays were performed using transwell migration chambers (8.0 µm pore inserts, BD Biosciences, USA). A total of 3 × 10^5^ cells with DMEM without FBS were seeded into the inner chamber, and DMEM medium with 10% FBS was added to the bottom chambers as an attractant. After 24 h of incubation, migratory cells on the lower surface were stained with 0.1% crystal violet solution (Sigma-Aldrich, USA), and colonies were counted immediately.

### Immunofluorescent staining

Cells were seeded in six-well pate, after washing with PBS and fixation with 4% paraformaldehyde. Then, the cells were permeabilized with 0.5% Triton X-100, and blocked in 10% goat serum at room temperature. Primary antibodies were incubated overnight, and the next day, cells were incubated with fluorescent secondary antibodies. Then, we imaged the nuclei of DAPI-labeled cells by confocal fluorescence microscopy (Nikon A1, Japan).

### Co-immunoprecipitation (Co-IP)

293T cells were collected and lysed using RIPA Lysis Buffer (Pierce, USA). Then, protein A/G beads (Santa Cruz, USA) were co-incubated with the primary antibody or IgG at 4 °C for 6 h. Cell lysate was added to the mixture of beads and antibody overnight at 4 °C. The beads–antibody–protein complexes were washed with precooled PBS solution three times (each time for 10 min). Finally, samples were detected by western blot analysis.

### Hematoxylin–eosin (HE) and immunohistochemistry (IHC) staining

For HE staining, sections were subjected to hematoxylin and eosin staining and observed with a light microscope (Leica, Japan). The representative images were taken by fluorescence microscope. Paraffin-embedded tissue sections were stained with an antibody against SPINK5 (Abcam, ab138511, USA), the slides were incubated with ABC (Vector Laboratories, USA) and overlaid with 3-30-diaminobenzidine (DAB; Dako, USA), and the nuclei were stained with hematoxylin, following a previously described method [21]. The slides were viewed using a BZ9000 microscope (Zeiss, Germany).

### In vivo tumor metastasis assay

Six- to eight-week-old SPF female C57BL/6 mice were purchased from the Beijing Laboratory Animal Center (Beijing, China) and randomly divided into three groups: (A) control group (*n* = 5), mice were treated with PBS after B16-F10 cell injection; (B) metformin treatment group, to measure the therapy function, mice were treated by 3 mg/kg of metformin for 14 days (intraperitoneally) after cell injection; (C) metformin prevent and treatment group, to evaluate the chemopreventive effects, mice were treated by 3 mg/kg of metformin for 7 days before and 14 days after cell injection. To establish a murine melanoma pulmonary metastasis model, 4 × 10^5^ B16-F10 cells were injected via the tail vein. Two weeks later, mice were sacrificed and lungs were carefully harvested, the tissues were fixed in a neutral-buffered formaldehyde solution, and the metastasis foci were counted as black or white forms on the tissue surface. This study was approved by the Experimental Animal Ethics Committee of the Third Affiliated Hospital of Kunming Medical University.

### Statistics

Data are presented as mean ± standard deviation (SD). SPSS for Windows version 18.0 (SPSS Inc., Chicago, IL, USA) was used for *χ*^2^ test and Student’s *t*-test. A *p*-value < 0.05 was considered significant.

## Results

### miR-5100 modulates melanoma EMT process

**Fig. 1:**
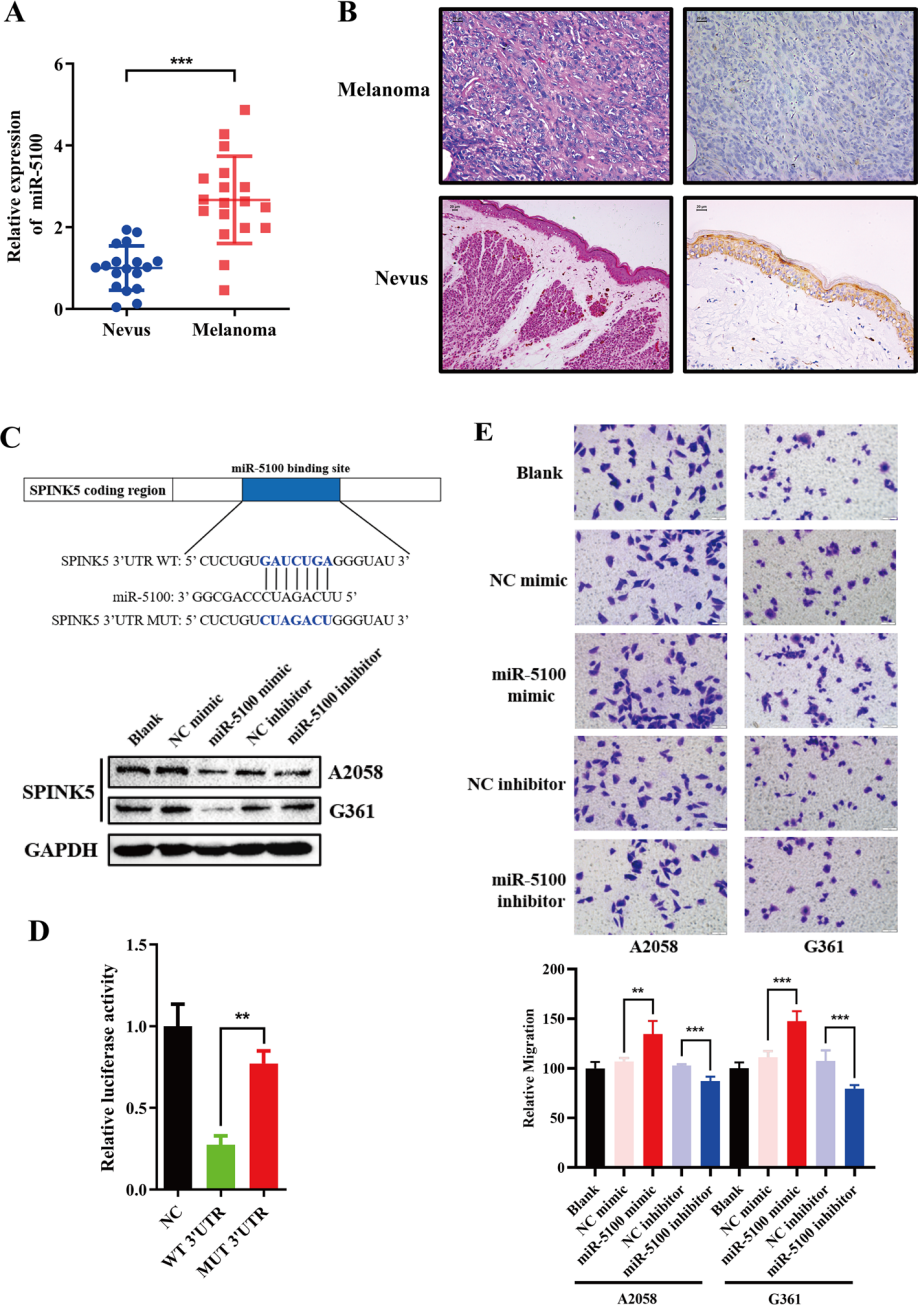

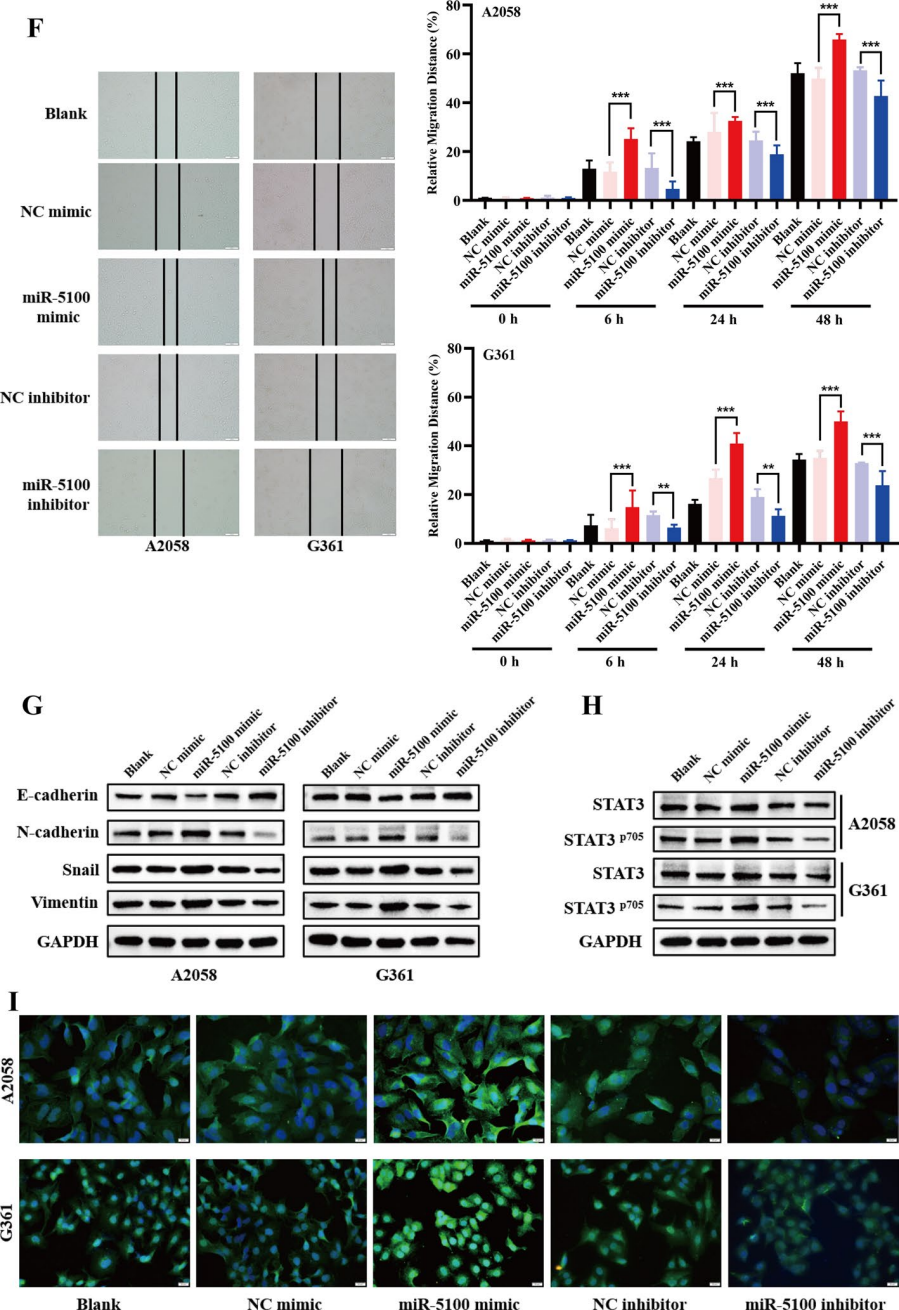
miR-5100 modulates melanoma cell EMT by targeting SPINK5–STAT3 pathway. **A**, **B** Expression of miR-5100 and SPINK5 was measured by RT-qPCR and IHC in melanoma and nevus specimens. **C**, **D** The miR-5100 binding site in the 3′-UTR of SPINK5 and matched mutations; miR-5100 modulates SPINK5 expression. Furthermore, a luciferase reporter assay was conducted in 293T cells to verify the interaction between miR-5100 and the SPINK5 binding site. **E**, **F** The metastasis inhibition effects of miR-5100 were measured by wound-healing and transwell assays. **G** miR-5100 modulates melanoma cell EMT process shown by EMT‑related markers determined by western blot analysis. **H**, **I** Immunoblotting and IF analysis of STAT3, and phosphorylation of STAT3 in A2058 and G361 cell lines transfected with either a mimic or an inhibitor of miR-5100. *p < 0.05, **p < 0.01, ***p < 0.001 versus control

To identify miRNA and mRNA differently expressed between melanoma and nevus, miRNA and mRNA expression microarray were employed (GSE183115, GSE183116), of which miR-5100 expression exhibited elevation in melanoma by qPCR (*p* < 0.001, Fig. 1A). On the contrary, IHC showed a lower expression of SPINK5 in melanoma (Fig. 1B, Tables 1, 2). Moreover, TargetScan (http://www.targetscan.org/) uncovered that SPINK5 is a potential target of miR-5100, and western blotting showed that the miR-5100 mimic decreased SPINK5 expression and the miR-5100 inhibitor elicited the opposite effect (Fig. 1C). Dual-luciferase reporter assays revealed that miR-5100 overexpression decreased the luciferase activity of pGL3-SPINK5-Wt but not that of pGL3-SPINK5-Mut (Fig. 1D). Furthermore, TCGA data revealed an increased expression of miR-5100 in metastatic melanoma, whereas SPINK5 exhibited the opposite trend by online bioinformatics analysis (http://ualcan.path.uab.edu/, data from TCGA, Additional file 2: Data S2).

**Table 1:** Clinicopathologic characteristics of patients with melanoma

Clinicopathologic characteristics	Number of samples (%)
Age, years	
< 50	18 (38.30)
≥ 50	29 (61.70)
Gender	
Male	30 (63.83)
Female	17 (36.17)
Ulceration	
Positive	23 (69.70)
Negative	10 (21.28)
Lymph node metastasis	
Positive	17 (36.17)
Negative	18 (38.30)
Pathologic grade	
I	10 (25.00)
II	13 (32.50)
III	14 (35.00)
IV	3 (7.50)

**Table 2:** Expression of SPINK5 in melanoma and nevus specimens

Disease	Total	SPINK5		*p*
		Positive (%)	Negative (%)	
Melanoma	47	16 (34.04)	31 (65.96)	0.001
Nevus	56	38 (67.86)	18 (32.14)	

*p* < 0.05, statistically significant difference

We then investigated the effect of miR-5100 on melanoma cell metastasis. Following transfection of melanoma cells with the miR-5100 mimic or inhibitor, wound-healing and transwell assays showed that miR-5100 mimic caused an obvious increase in migration cells of A2058 and G361 compared with the control group, while miR-5100 inhibitor had the opposite effect (Fig. 1E, F).

We next focused on identifying a mechanistic basis for how miR-5100 promotes a metastatic phenotype. Studies have reported that EMT has implications for tumor cell metastasis by triggering the loss of cell–cell adhesion [24]. The levels of EMT makers were examined by western blot. The expression of epithelial marker E-cadherin was decreased while mesenchymal markers N-cadherin, Snail, and Vimentin expression levels increased after miR-5100 mimic transfection; meanwhile, the opposite result was observed after the interference of miR-5100 inhibitor (Fig. 1G).

It has been shown that the initiation of EMT was mostly modulated by STAT3 activation [25]. In this study, miR-5100 mimic promoted STAT3 expression and phosphorylation at tyrosine 705 (Fig. 1H, I). These data suggest that miR-5100 modulates melanoma EMT probably via the SPINK5–STAT3 pathway.

### SPINK5–STAT3 pathway modulates melanoma EMT process

**Fig. 2:**
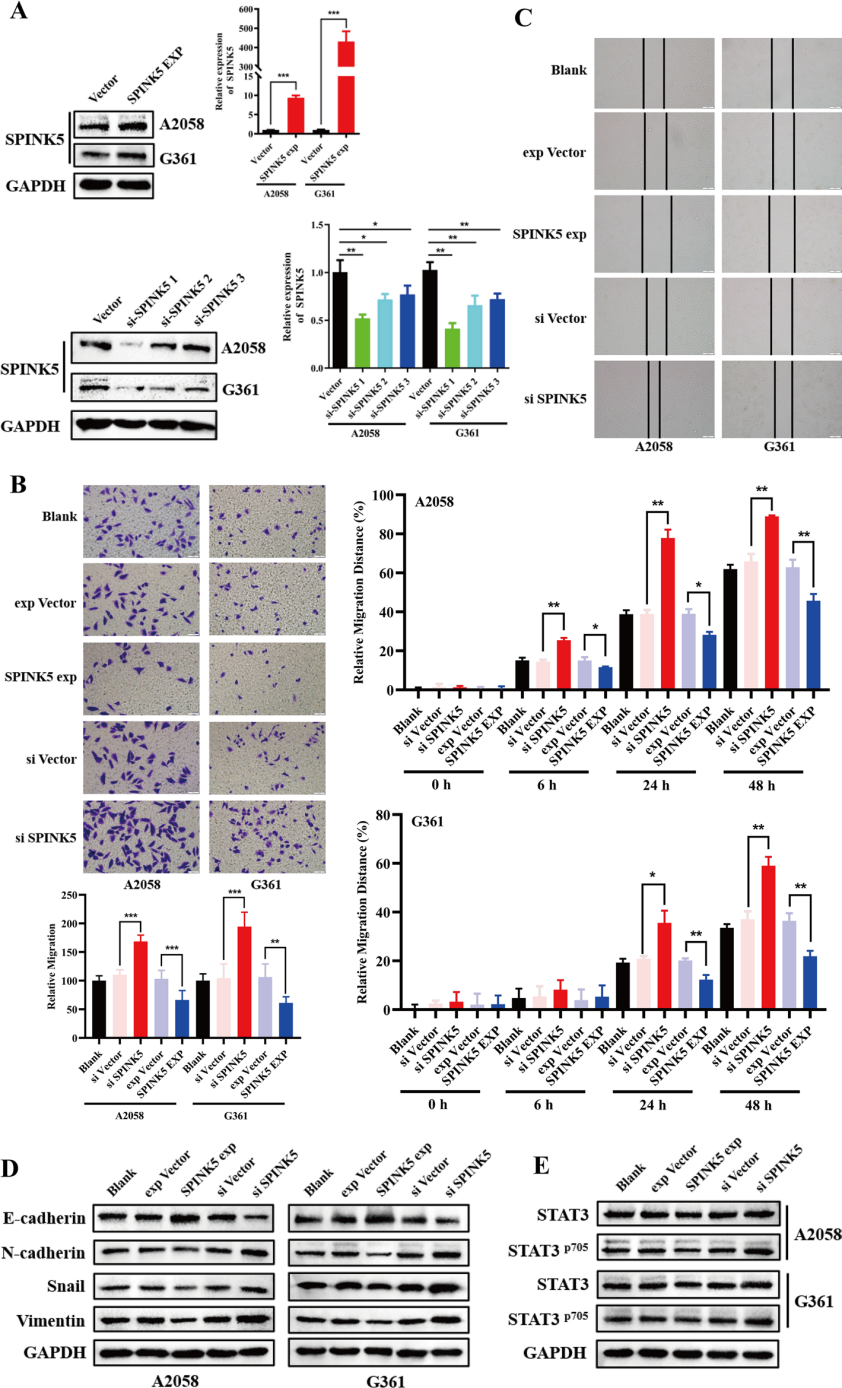

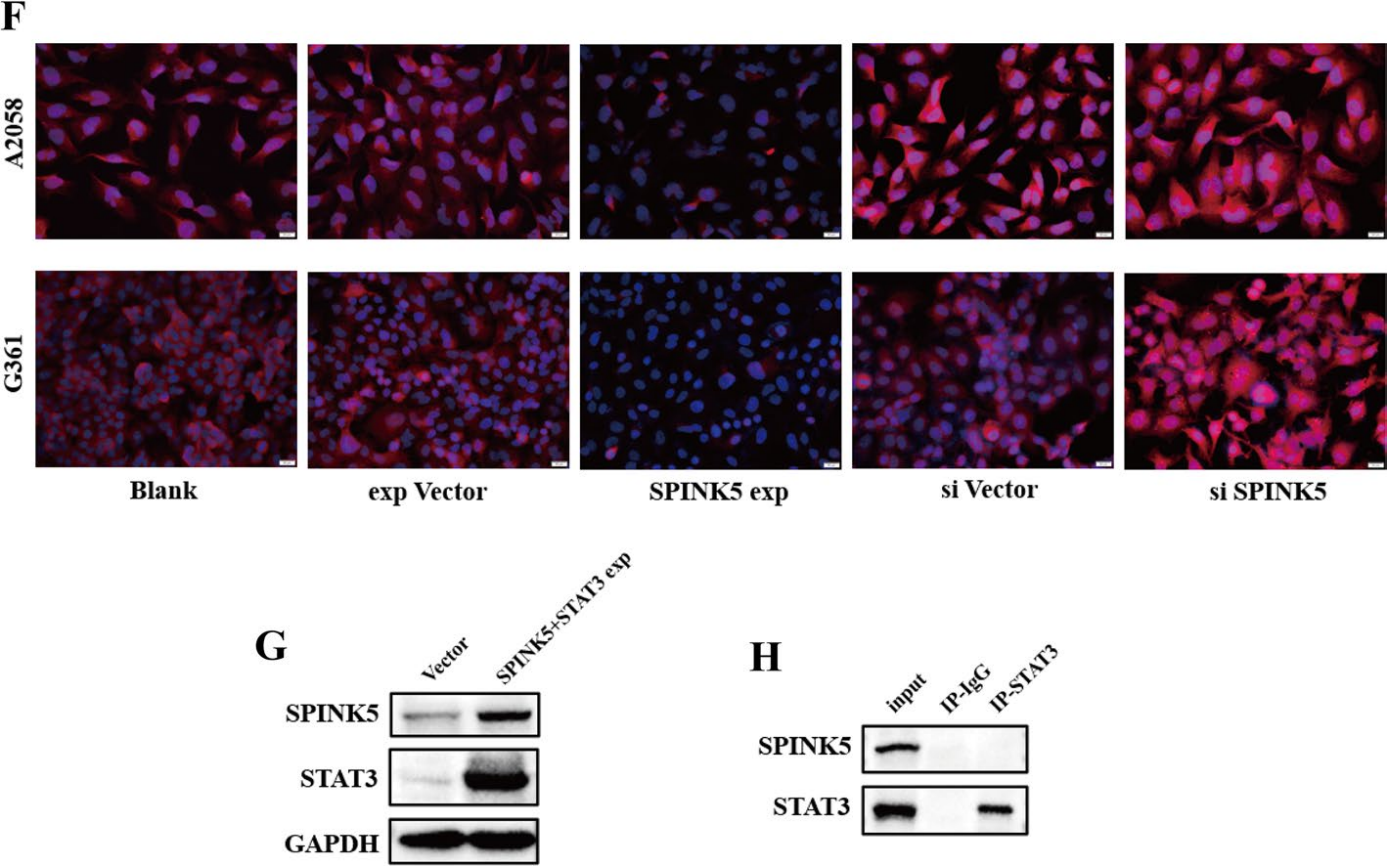
SPINK5 modulates melanoma cells EMT by STAT3 phosphorylation. **A** Western blot and RT-qPCR were used to detect SPINK5 expression after transfection with either an siRNA or an expression vector. **B**, **C** Wound-healing and transwell assays were used to determine the effects of SPINK5 in melanoma cell metastasis. **D** SPINK5 modulates melanoma cell EMT process shown by EMT‑related markers determined by western blot analysis. **E**, **F** Immunoblotting and IF analysis of STAT3, and the phosphorylation of STAT3 in A2058 and G361 cell lines transfected with either a SPINK5 siRNA or expression vector. **G**, **H** After cotransfection, Co-IP was used to measure the interaction between SPINK5 and STAT3 in 293T cells. *p < 0.05, **p < 0.01, ***p < 0.001 versus control

To explore the functions of SPINK5 in melanoma cells, its effects on the migration of A2058 and G361 cells was examined. We found that knockdown of SPINK5 apparently elevated the metastasis ability of melanoma cells. Conversely, SPINK5 expression inhibited cells’ metastasis capacity (Fig. 2A–C). In line with the cell motility change, knockdown of SPINK5 decreased E‑cadherin protein expression but increased mesenchymal markers. As expected, SPINK5 overexpression induced a mesenchymal–epithelial transition phenotype (Fig. 2D).

STAT3 is one of the pivotal proteins involved in the EMT process. The present study uncovered that SPINK5 knockdown induced STAT3 phosphorylation and nucleus location, and contrary findings were observed after SPINK5 expression vector transfection (Fig. 2E, F). Moreover, after cotransfection, we performed Co-IP assay to verify the interaction between SPINK5 and STAT3. IgG (negative control) blocked the antigen–antibody binding reaction. In the input (positive control), we detected STAT3 and SPINK5 by western blot, but in the IP group only STAT3 was detected. These findings confirm that SPINK5 cannot interact with STAT3 (Fig. 2G, H) and indicate that SPINK5 modulates melanoma EMT by STAT3, indirectly.

### Metformin inhibits EMT by regulating the miR-5100/SPINK5/STAT3 axis

**Fig. 3:**
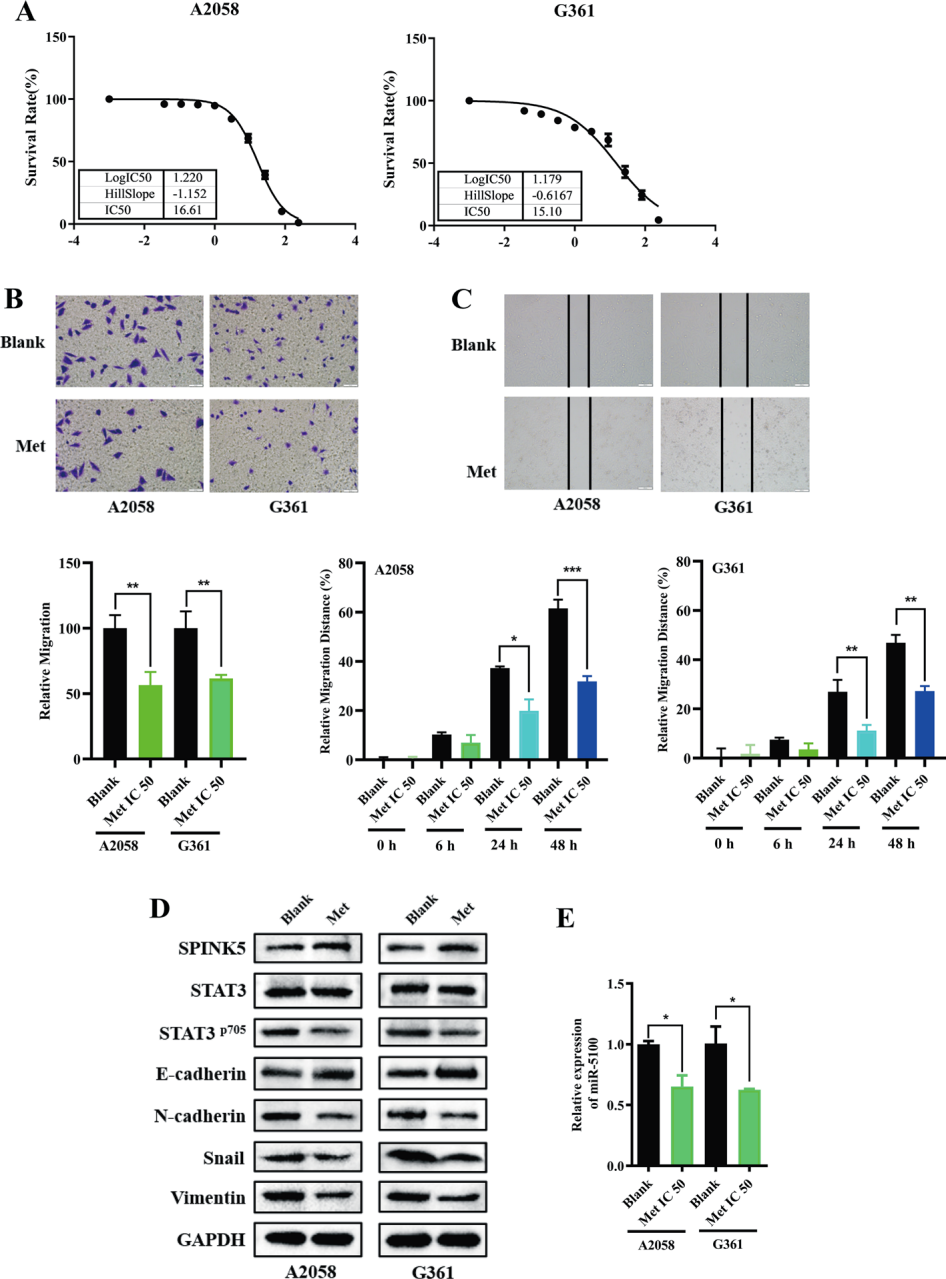

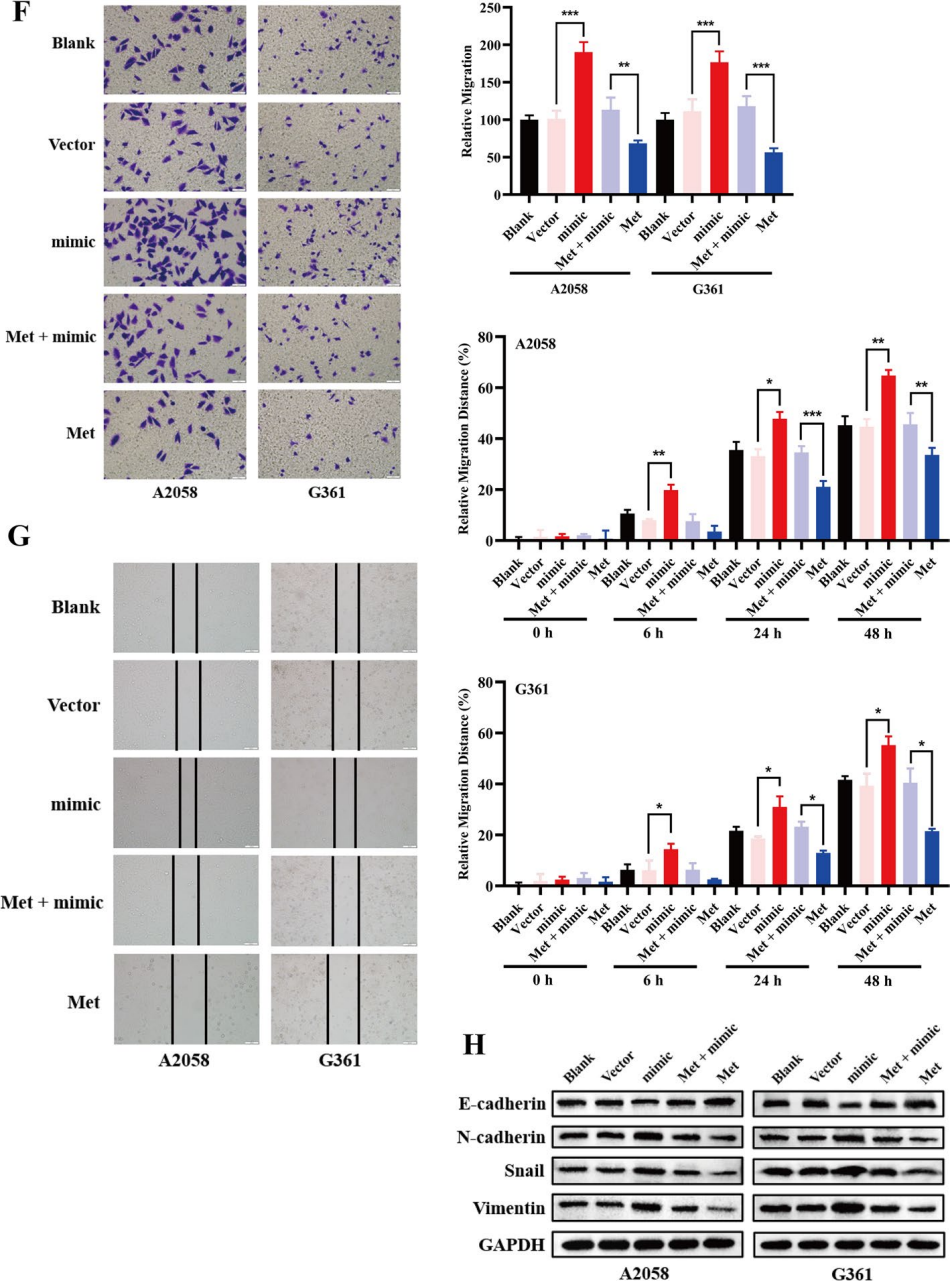
Metformin inhibits EMT by regulating the miR-5100/SPINK5/STAT3 axis. **A** IC_50_ were detected in A2058 and G361 cells (16.61 and 15.1 mM, respectively). **B**, **C** Wound-healing and transwell assays were used to determine the effects of metformin in melanoma cell metastasis. **D**, **E** Western blot and RT-qPCR were used to detect miR-5100 and relative protein expression after treatment with metformin under a concentration of IC_50_. **F**–**H** miR-5100 mimic reversed metastasis and EMT inhibition effect of metformin in melanoma cells partly. *p < 0.05, **p < 0.01, ***p < 0.001 versus control

The antitumor activity toward melanoma cells was evaluated for metformin. IC_50_ values were obtained from the MTT assay. In this experiment, metformin exhibited IC_50_ values of 16.61 and 15.10 mM for A2058 and G361 cells, respectively, at 48 h (Fig. 3A). On the basis of the IC_50_ results, the antimetastatic effect of metformin on melanoma cells was examined by wound-healing and transwell assays. As demonstrated, metformin significantly inhibited the motility of the melanoma cells (Fig. 3B, C). Meanwhile, metformin treatment reduced mesenchymal cell markers, including Snail, Vimentin, and N-cadherin, but restored E-cadherin expression (Fig. 3D), indicating that metformin inhibited migration of melanoma cells by reversing the EMT process.

Then, to explore the potential mechanism by which metformin mediated inhibition of the metastasis of melanoma cells, the expression of miR-5100, SPINK5, and STAT3 was detected after treatment with metformin for 48 h. As shown, metformin inhibited expression of miR-5100 and pSTAT3 but upregulated SPINK5 (Fig. 3D, E). Moreover, miR-5100 mimic neutralized the inhibitory effect of metformin on cell metastasis and EMT process partly (Fig. 3F–H).

These data indicate that metformin suppressed melanoma cell migration by regulating the miR-5100/SPINK5/STAT3 axis.

### Metformin inhibits melanoma cells metastasis in vivo

**Fig. 4:**
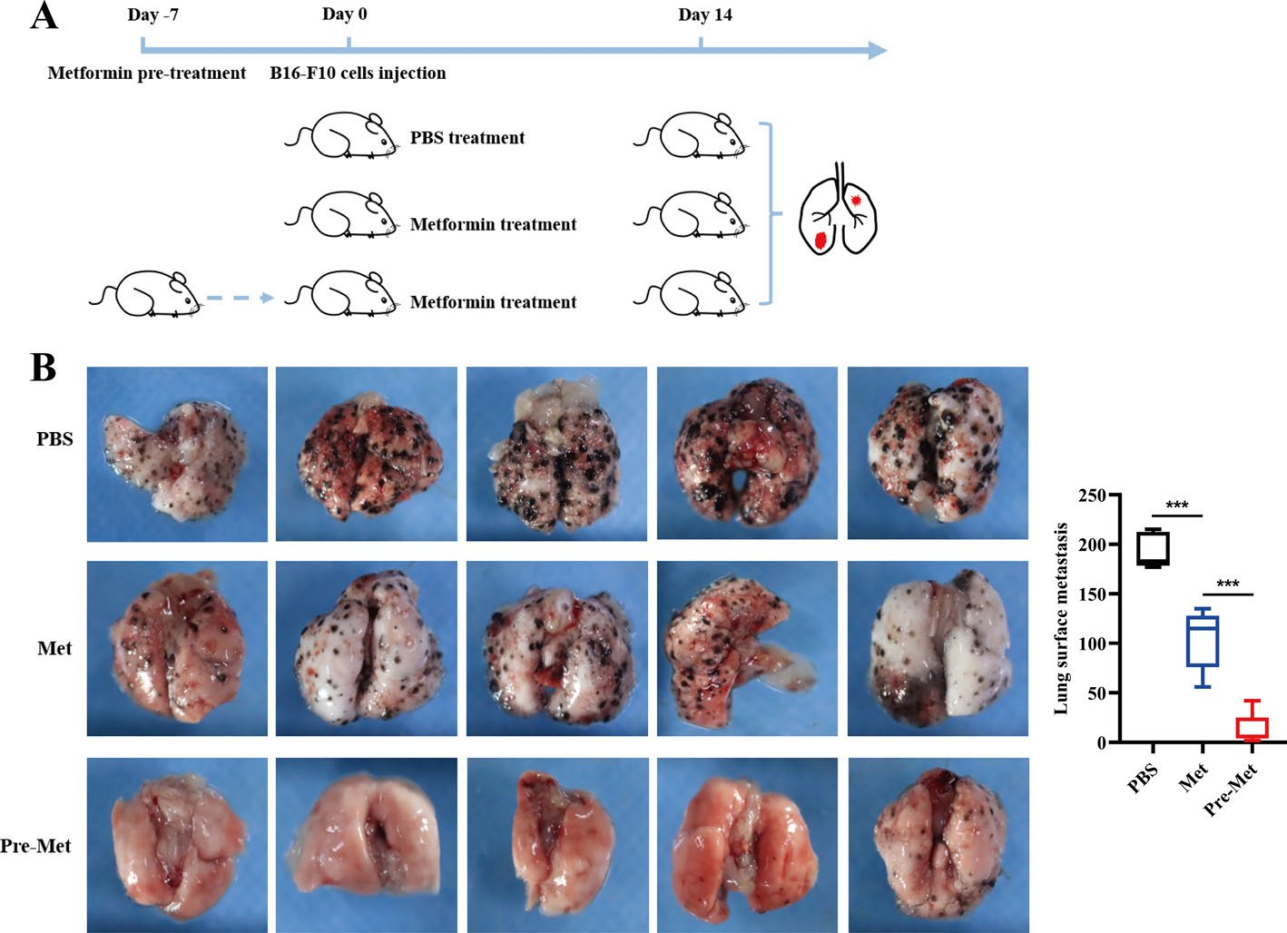
Metformin inhibits melanoma cells metastasis in vivo. **A**, **B** Metformin suppressed pulmonary metastasis. Pretreatment of metformin inhibited melanoma cell metastasis to lung more effectively. *n* = 5. The black dots on the surface of the lungs indicate metastasis foci. *p < 0.05, **p < 0.01, ***p < 0.001 versus control

To corroborate our in vitro results, C57 mice were injected with B16F10 cells (1.0 × 10^6^ cells per mouse) into the tail vein. To confirm that metformin served as a metastasis inhibitor for B16F10 tumor cells, 14 days after melanoma cell injection, all mice were sacrificed and lung was removed to count the metastasis foci. Moreover, we treated mice with metformin before B16F10 injection (Fig. 4A). Notably, we found that metformin restored the metastatic capacity of B16F10. Interestingly, metformin pretreatment improved this effect further (Fig. 4B).

## Discussion

On the basis of high-throughput microarray technology, we acquired amount of differently expressed miRNAs. In the present study, we focused on molecules participating in melanoma metastasis regulation. Integrating our results with TCGA data, we found that miR-5100 expression was upregulated in melanoma tissues. In concordance with previous studies [26, 27], our results demonstrated that miR-5100 could modulate melanoma cells’ EMT process. miRNA could regulate many different target mRNAs, according to our microarray results and TCGA database, and we uncovered that SPINK5 expression is opposite to that of miR5100. Furthermore, TargetScan also indicated a regulative relation between these two molecules. In this study we confirmed that SPINK5 served as a direct target of miR-5100.

SPINK5, a serine protease inhibitor that contains 15 Kazal-type serine protease inhibitory domains [28], has been considered as a tumor suppressor in various cancers [29, 30]. Moreover, SPINK5 was considered as marker for prediction of lymph node metastasis in HNSCC [31]. Herein, we found that SPINK5 was remarkably decreased in melanoma compared with nevus tissues, suggesting that SPINK5 may be a potential tumor suppressor in melanoma. Furthermore, silencing of SPINK5 prominently increased the metastasis abilities of melanoma cells, whereas SPINK5 overexpression exhibited the opposite trend. These were further confirmed by measurement of EMT markers. Thus, our data suggest that SPINK5 inhibits melanoma cells motility by modulating the EMT process.

EMT is considered to be a critical event in cancer cell migration, which is driven by the STAT3 pathway [32]. STAT3 activity is dependent on two phosphorylation sites: phosphorylation of tyrosine 705 (Try 705) and serine 727 (Ser 727). Recently, a study revealed that the pY705–STAT3 pathway elicits EMT and pS727–STAT3 signaling induces MET process [16].

Mechanistically, activated Janus kinase (JAK) leads to an increase in STAT3 phosphorylation at Y705 [33]. It has been confirmed that expression of Kallikrein-related peptidases (KLKs) could induce the activation of JAK [34]. KLKs constitute a large family of secreted trypsin and chymotrypsin-like serine proteinases that are expressed in multiple tissues [35]. However, this process is inhibited by SPINK5 directly [36]. In concordance with this, our results confirmed that SPINK5 expression inhibited STAT3 Tyr705 phosphorylation. Disappointingly, Co-IP demonstrated an indirect interaction between SPINK5 and STAT3. Thus, further study is required to uncover the underlying modulation mechanisms between SPINK5 and STAT3.

Metformin has been revealed to serve as a potent antitumor drug for the treatment of multiple cancers, including melanoma [21, 37–39]. Some cohort studies have shown that metformin suppresses the invasion and migration of various types of cancer [40, 41]. Researchers have documented that metformin blocks melanoma cell invasion and metastasis through AMPK activation [42], which decreases cancer cell mTOR signaling and protein synthesis [43]. We have proven that metformin could inhibit the proliferation and stemness of NSCLC [21]. Moreover, studies have uncovered that melanoma cell growth and motility were hampered by metformin treatment through modulation of various microRNA expression [44]. However, the underlying antimetastatic mechanism of metformin on the regulation of miRNAs in melanoma remains unclear. Thus, we employed metformin to measure its antimetastatic function. We show here that metformin treatment inhibited melanoma cell migration and EMT process. Notably, metformin suppressed miR-5100 but elevated SPINK5 expression, importantly, which inhibited STAT3 expression and Tyr705 phosphorylation. However, the underlying mechanisms by which metformin modulates miR-5100 expression remain to be discussed.

Studies have shown that patients with diabetes who had taken metformin had a reduction of incidence and mortality of various cancers [38, 39, 45]. Moreover, it is reported that metformin could reduce the risk of metastasis in breast cancer [46, 47]. Consistently, our in vivo study showed that metformin suppressed melanoma cell metastasis. More importantly, metformin pretreatment abolished the pulmonary metastasis of B16-F10 cells. We suspect metformin pretreatment not only impacts intracellular pathways to inhibit metastasis, but also modulates the extracellular microenvironment, e.g., adhesion molecules [48], vascular leakiness [49], and extracellular matrix remodeling [49] in distant organs. Of note, metformin also led to the activation of antitumor immune and metabolic reprogramming in both lab experiments and clinical trials [50–53]. Thus, metformin pretreatment reinforced these effects and showed fewer metastasis foci.

As the metastasis foci are too tiny and scattered to be collected for further study, we failed to provide in vivo evidence that metformin modulates miR-5100, SPINK5, STAT3, and EMT protein expression. However, studies have uncovered that metformin reduced the number of metastases in animal models [41]. Moreover, it has been confirmed that metformin could inhibit EMT protein expression in a xenograft mouse model bearing tumors [54], decrease the circulating tumor cell (CTC) adhesion to activated endothelial cells, and alleviate lung vascular permeability [55]. These findings provide indirect evidence that metformin inhibits lung metastasis by modulating the EMT process in our in vivo model.

In summary, metformin suppressed melanoma cell migration and EMT. Moreover, this effect is partly dependent on the miR-5100/SPINK5/STAT3 pathway, indicating that metformin is a potential oncotherapeutic agent. These findings may provide a possible strategy for the clinical treatment of melanoma.

## Availability of data

We declare that the materials described in the manuscript, including all relevant raw data, will be freely available to any scientist wishing to use them for noncommercial purposes, without breaching participant confidentiality.

## Supplementary Information

**Additional file 1: Data S1.** Primer sequences for RT-qPCR. Clinicopathologic characteristics of melanoma and nevus patients.

**Additional file 2. Data S2.** TCGA data showed the expression patterns of miR-5100 and SPINK5 in normal tissue, primary melanoma and metastic melanoma.

## Abbreviations

EMT: Epithelial–mesenchymal transition
SPINK5: Serine peptidase inhibitor Kazal type 5
STAT3: Signal transducer and activator of transcription 3
RT-PCR: Quantitative polymerase chain reaction
Co-IP: Co-immunoprecipitation
HE: Hematoxylin–eosin
IHC: Immunohistochemistry
