# Supplementary material for: Metformin inhibits melanoma cell metastasis by suppressing the miR-5100/SPINK5/STAT3 axis
Source: Cell Mol Biol Lett. 2022 Jun 15;27:48. doi: 10.1186/s11658-022-00353-5 (PMC9199130; doi:10.1186/s11658-022-00353-5)
Supplement: Supplementary file 1 — Additional file 1: Data S1. Primer sequences for RT-qPCR. Clinicopathologic characteristics of melanoma and nevus patients. [file 11658_2022_353_MOESM1_ESM.docx]

Additional file 1: Data S1

Primer sequences for RT-qPCR.

| Target | Primer sequence (5′-3′) | |
| --- | --- | --- |
| SPINK5 | Forward: | 5’- GCCTCATACAAGATGCTGCC -3’ |
|  | Reverse: | 5’- TAAATGCCTGGCCCTCTTCT -3’ |
| STAT3 | Forward: | 5’- CAGTGACCAGGCAGAAGA -3’ |
|  | Reverse: | 5’- ACTCCATCGCTGACAAAA -3’ |
| GAPDH | Forward: | 5’-TCAAGAAGGTGGTGAAGCAGG -3’ |
|  | Reverse: | 5’-TCAAAGGTGGAGGAGTGGGT -3’ |
| miR-610 | Forward: | 5’- TGCGCTTCAGATCCCAGCGGTG -3’ |
|  | Reverse: | 5’-CAGTGCGTGTCGTGGAGT-3’ |
| U6 | Forward: | 5’-GCTTCGGCAGCACATATACTAAAAT-3’ |
|  | Reverse: | 5’-CGCTTCACGAATTTGCGTGTCAT-3’ |

Clinicopathologic characteristics of melanoma and nevus patients.

| Clinicopathologic characteristics | Melanoma (%) | Nevus (%) | *p* value |
| --- | --- | --- | --- |
| Age |  |  |  |
| <50 | 11 (61.11) | 10 (55.56) | *p* > 0.05 |
| ≥50 | 7 (38.89) | 8 (44.44) |  |
| Gender |  |  |  |
| Male | 12 66.67) | 10 (55.56) | *p* > 0.05 |
| Female | 6(33.33) | 8 (44.44) |  |
| Race |  |  |  |
| Han | 18 (100) | 18 (100) | *p* > 0.05 |
| Other | 0 (0) | 0 (0) |  |
| Pathologic grade |  |  |  |
| I | 3 (16.67) | N/A | N/A |
| II | 7(38.89) |  |  |
| Ⅲ | 7(38.89) |  |  |
| Ⅳ | 1(5.50) |  |  |
