# Supplementary material for: Metformin inhibits melanoma cell metastasis by suppressing the miR-5100/SPINK5/STAT3 axis
Source: Cell Mol Biol Lett. 2022 Jun 15;27:48. doi: 10.1186/s11658-022-00353-5 (PMC9199130; doi:10.1186/s11658-022-00353-5)
Supplement: Supplementary file 2 — Additional file 2. Data S2. TCGA data showed the expression patterns of miR-5100 and SPINK5 in normal tissue, primary melanoma and metastic melanoma. [file 11658_2022_353_MOESM2_ESM.pdf]

Supplementary data 2

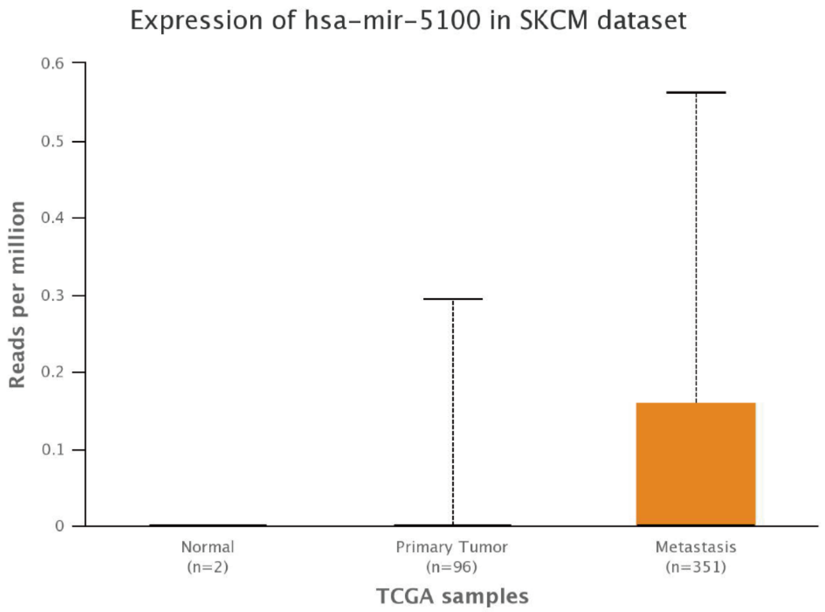

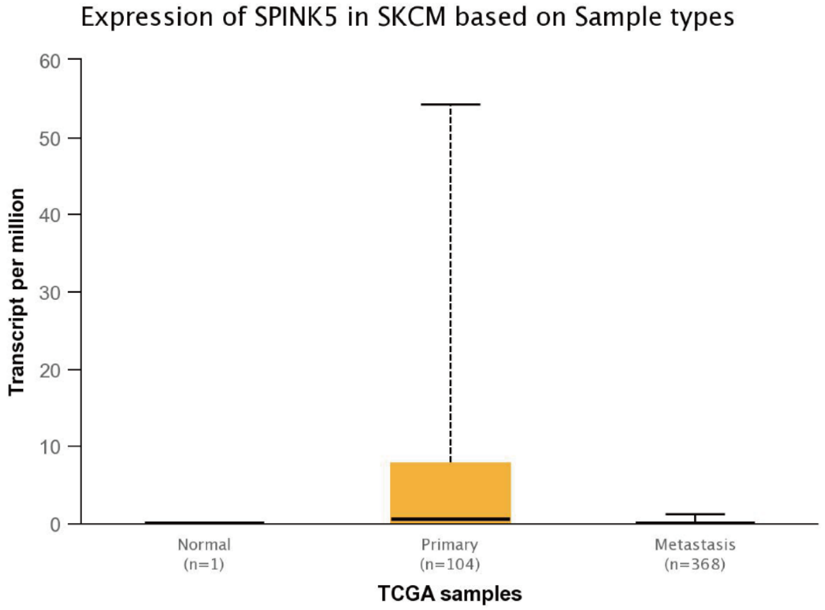

TCGA data showed the expression patterns of miR-5100 and SPINK5 in normal tissue, primary melanoma and metastic melanoma.
